# A Case of Vasculitic Neuropathy Presenting With Progressive Sensory-Motor Limb Involvement, Posterior Reversible Encephalopathy Syndrome, and Systemic Symptoms in a 70-Year-Old Woman

**DOI:** 10.7759/cureus.96058

**Published:** 2025-11-04

**Authors:** Kushal Panja, Syed Osama Husain, Deepa Bai, Daniel Du Plessis, Rajeev Upreti, Bushra Jamil

**Affiliations:** 1 General Medicine, North Manchester General Hospital, Manchester University NHS Foundation Trust, Manchester, GBR; 2 Cellular Pathology, Salford Royal Hospital NHS Foundation Trust, Salford, GBR

**Keywords:** axonal neuropathy, nerve biopsy, posterior reversible encephalopathy syndrome (pres), severe hypertension, vasculitic neuropathy

## Abstract

We present the case of a 70-year-old woman with progressive weakness and altered sensation in her hands and feet, complicated by intermittent confusion, status epilepticus, and posterior reversible encephalopathy syndrome (PRES). She was known to have cervical spinal stenosis and gave a vague history of an autoimmune disorder, which was in remission for the past many years. Extensive evaluation for infectious, compressive, and paraneoplastic causes was unrevealing. Investigations revealed severe axonal peripheral neuropathy with active vasculitic features confirmed on nerve biopsy, which were not picked up by the PET scan. With aggressive blood pressure control and high-dose corticosteroids, PRES resolved radiologically, and neurological function improved gradually. This case highlights the diagnostic complexity of systemic vasculitis presenting with simultaneous central and peripheral nervous system involvement, underscores the indispensable role of biopsy when non-invasive tests are inconclusive, and illustrates how timely immunosuppression and hemodynamic control can reverse central manifestations while stabilizing peripheral nerve injury.

## Introduction

Vasculitic neuropathy is an uncommon but serious manifestation of systemic vasculitis that can present with painful, asymmetric sensorimotor deficits (often mononeuritis multiplex) and may lead to permanent disability if treatment is delayed [1-3]. Electrodiagnostic testing typically shows an axonal pattern; however, histopathology remains the diagnostic gold standard because serologic and imaging findings can be non-specific or even normal in a substantial proportion of patients [1-4]. Contemporary reviews and systematic analyses indicate that sural (or superficial peroneal) nerve biopsy has moderate sensitivity, and that adding a muscle biopsy can modestly increase diagnostic yield when clinical suspicion remains high [2,5,6]. However, as demonstrated in this case, a nerve biopsy alone may be sufficient when classical vasculitic features are present in the biopsy.

Posterior reversible encephalopathy syndrome (PRES) is a clinico-radiologic entity characterized by seizures, encephalopathy, and visual symptoms with MRI evidence of vasogenic edema, usually precipitated by acute hypertension, renal dysfunction, cytotoxic or immunomodulatory therapies, and systemic inflammatory states [7-9]. PRES can complicate autoimmune and vasculitic disease--even early in the disease course--reflecting widespread endothelial dysfunction and impaired vascular autoregulation [10]. This case demonstrates that systemic vasculitis can simultaneously involve both the central and peripheral nervous systems.

## Case presentation

A 70-year-old woman was brought to the accident and emergency department after developing progressive weakness and altered sensation in both her hands and feet. She reported being unable to bear weight and, a day before admission, had experienced a sudden episode of urinary incontinence. Alongside these neurological complaints, she described a gradual decline in her health over the past nine weeks, marked by unintentional weight loss of approximately one stone, occasional nausea and vomiting, and a sense of malaise. For the past month, her family had also noticed episodes of intermittent confusion.

She had documented cervical spinal stenosis and, a month earlier, a flare-up of Raynaud's phenomenon associated with painful arthritis in her hands, swelling, and skin discoloration. She also had longstanding osteoarthritis, fibromyalgia, and rheumatoid arthritis, though the latter had been in remission for years without the need for immunosuppressive therapy.

On initial assessment, her hemoglobin was 75 g/L, and she was transfused with one unit of packed red blood cells. Blood tests confirmed chronic iron deficiency anemia with a low mean corpuscular volume, while vitamin B12 and folate levels were normal (Table 1).

**Table 1:** Lab values showing iron deficiency anemia.

Parameters	Lab values	Reference range
Hemoglobin	75	115.0–165.0 g/L
Mean corpuscular volume (MCV)	74.90	80.00–98.00 fL
Mean corpuscular hemoglobin concentration (MCHC)	318	320–365 g/L
Iron	2.6	5.8–34.5 umol/L
Ferritin	187	15–150 ug/L
Transferrin	1.71	2.00–3.60 g/L
Transferrin saturation	6.1	15.0–45.0%
Serum vitamin B12	700	197–771 ng/L
Folate	4.2	3.9–20.0 ug/L

**Figure 1:**
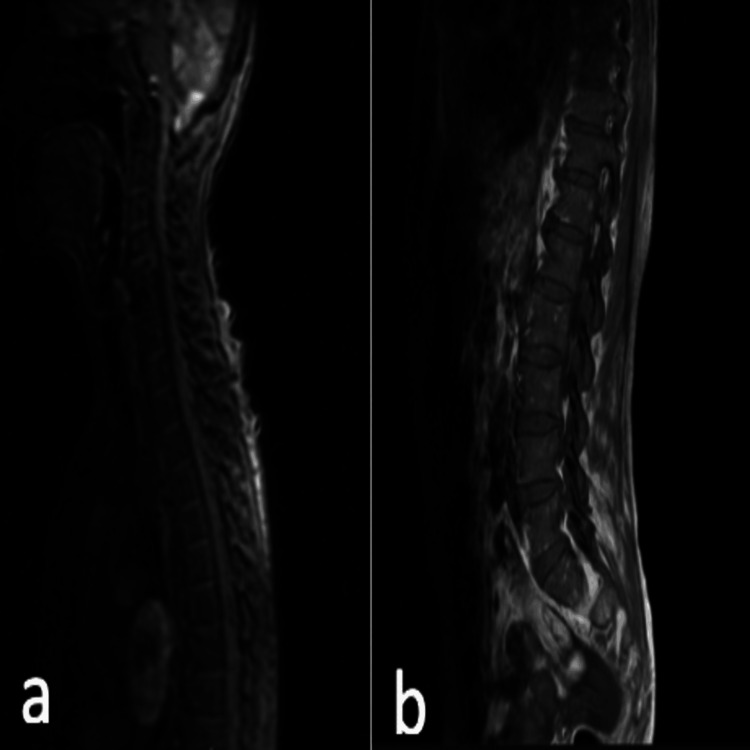
T1 and sagittal MRI spine showing multilevel degenerative spondylotic changes in the cervical and lumbar spine. (a) Cervical MRI and (b) lumbar MRI.

**Figure 2:**
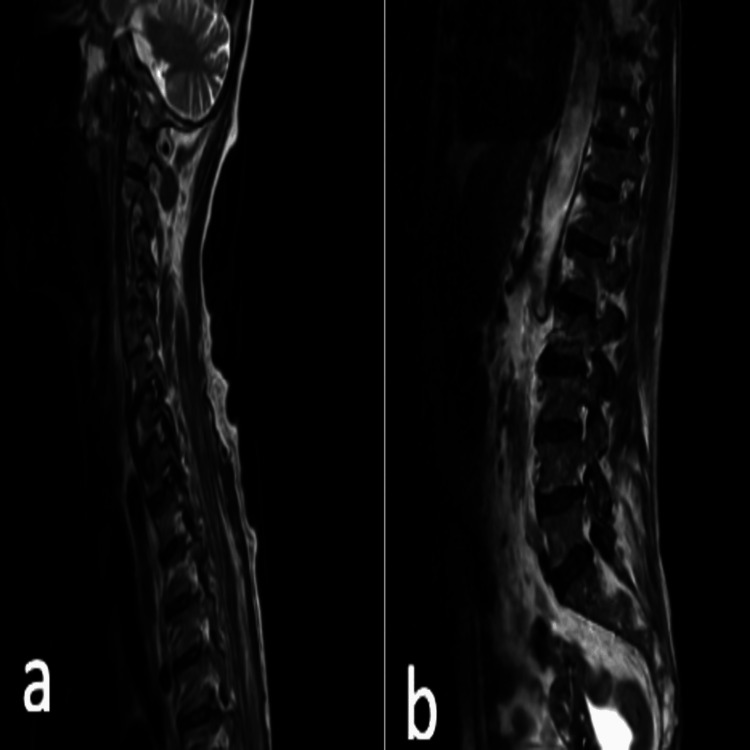
T2 sagittal MRI showing multilevel degenerative spondylotic changes in the cervical and lumbar spine. (a) Cervical MRI and (b) lumbar MRI.

Given her history of cervical disease, an urgent MRI of the spine was performed. This showed multilevel degenerative spondylotic changes in the cervical and lumbar spine, with disc herniations at several levels. A posterior disc herniation at C4/C5 caused indentation on the thecal sac and the anterior aspect of the cord with mild foraminal narrowing. Importantly, there was no evidence of acute spinal canal stenosis, cord or cauda equina compression, or myelopathy, effectively ruling out cord compression as the cause of her acute neurological symptoms (Figures 1, 2).

**Figure 3:**
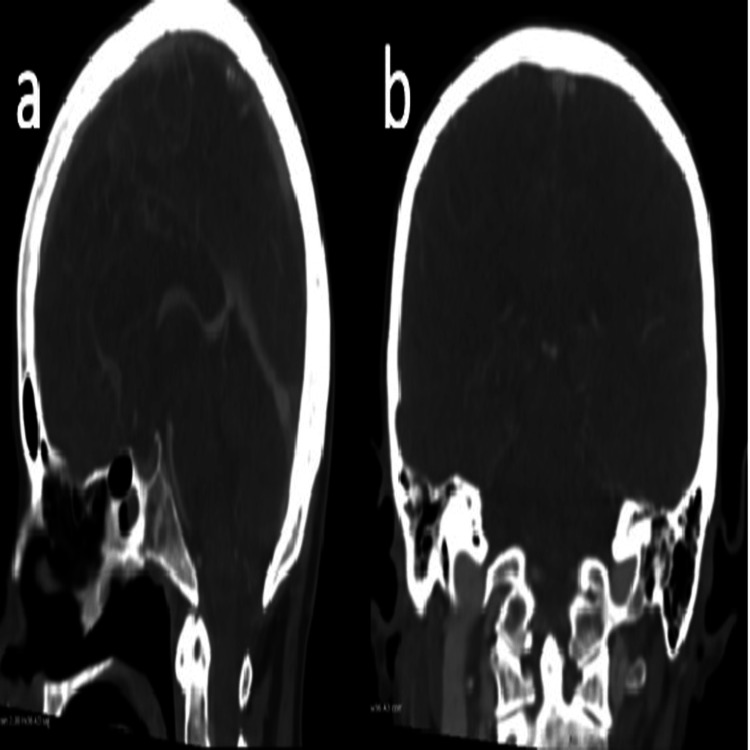
CT venogram of the head showing normal intracranial venous vasculature with no evidence of venous sinus thrombosis. (a) Sagittal view and (b) coronal view.

**Figure 4:**
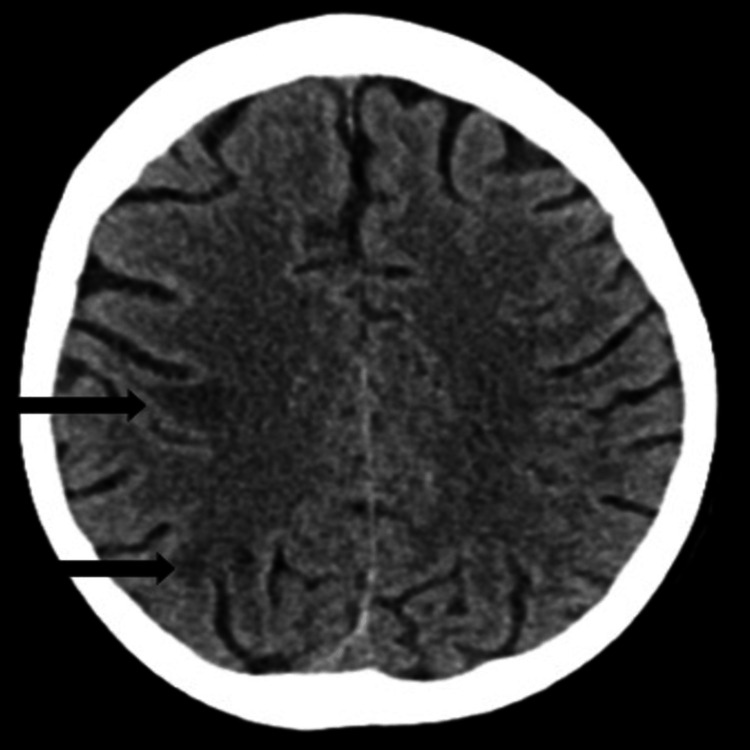
Non-contrast CT head (axial plane) revealing radiological features suspicious for posterior reversible encephalopathy syndrome (PRES).

**Figure 5:**
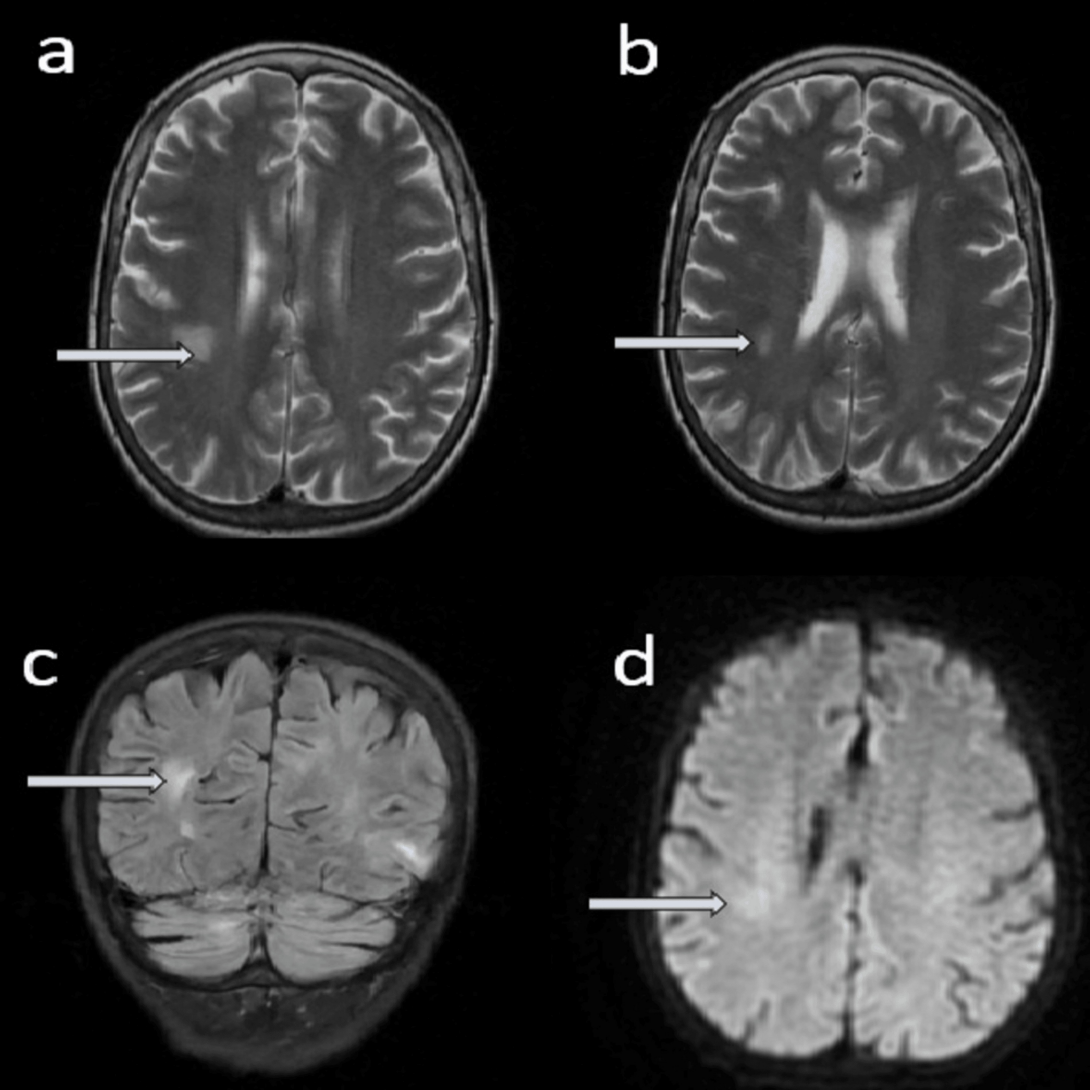
MRI head without contrast revealing radiological features of posterior reversible encephalopathy syndrome (PRES). (a) and (b) T2W images-axial section, (c) FLAIR view-coronal section, and (d) DWI image-axial section. DWI: diffusion-weighted imaging; FLAIR: fluid-attenuated inversion recovery.

The following day, her condition suddenly deteriorated. She developed status epilepticus, low Glasgow Coma Scale (GCS) (E2V3M5 -10/15), and her blood pressure at the time was documented at 200/80 mmHg. She required transfer to intensive care for seizure control and blood pressure management. A CT venogram of the head showed normal intracranial venous vasculature with no evidence of venous sinus thrombosis (Figure 3). A non-contrast CT head revealed radiological features suspicious for posterior reversible encephalopathy syndrome (PRES) (Figure 4), which was later confirmed by MRI of the brain (Figure 5). This diagnosis provided a partial explanation for her seizures and fluctuating confusion.

Initially, meningoencephalitis was considered, and she was empirically treated with ceftriaxone, amoxicillin, and acyclovir. However, cerebrospinal fluid analysis revealed normal glucose, mildly elevated protein, a white cell count of 5, and no organisms on Gram stain or culture. Blood cultures remained negative, and antimicrobial therapy was stopped.

**Figure 6:**
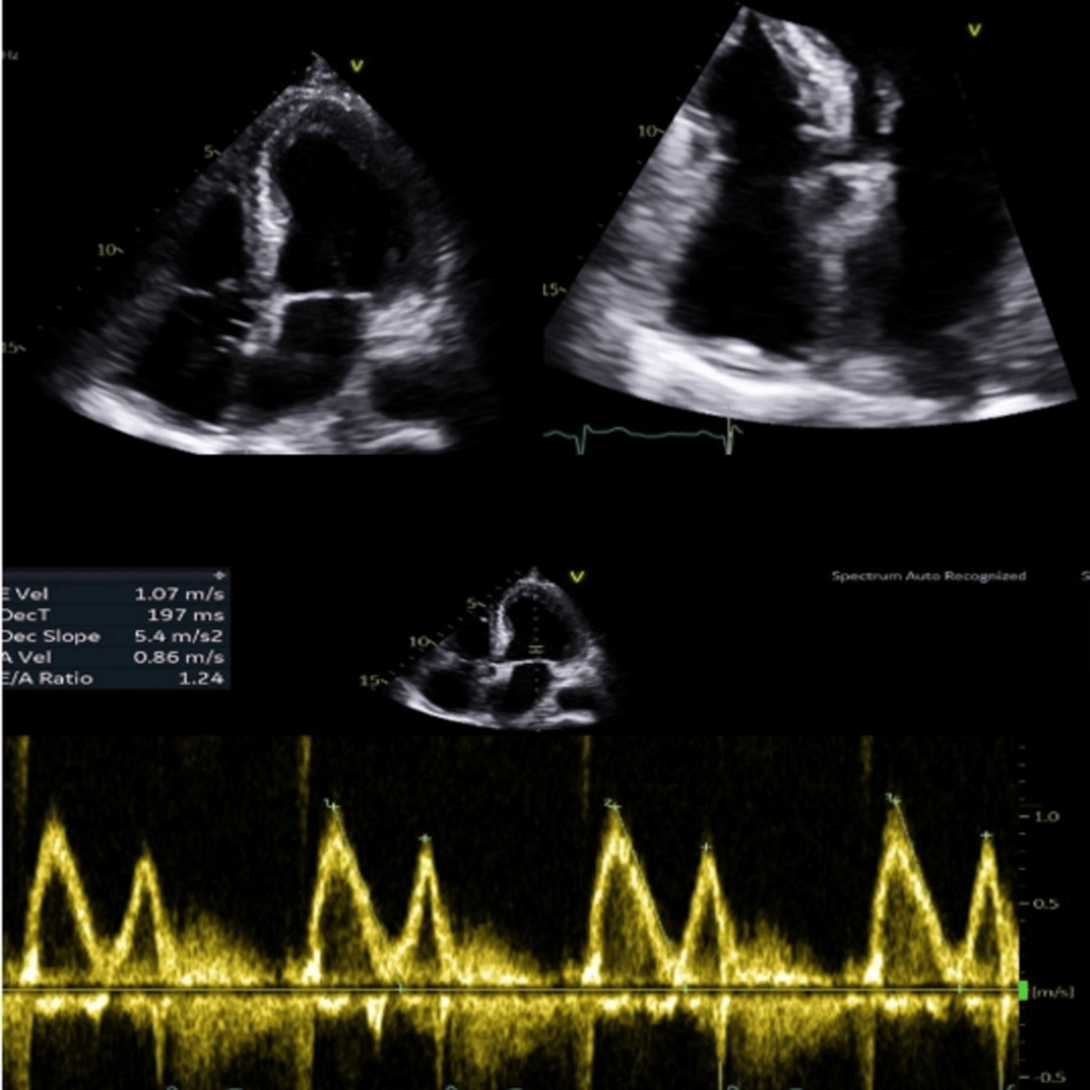
Echo images showing mild pericardial effusion.

Despite aggressive antihypertensive therapy, her blood pressure remained difficult to control, necessitating ongoing ICU support. Echocardiography demonstrated preserved left ventricular systolic function, mild tricuspid and mitral regurgitation, mild pulmonary hypertension, a left pleural effusion, and a trivial pericardial effusion without hemodynamic compromise (Figure 6). These findings were thought to be reactive, and cardiology advised no immediate intervention.

As her condition stabilized, attention shifted back to the underlying cause of her neurological decline. Differential diagnoses, including paraneoplastic syndromes, systemic vasculitides, and lupus, were considered. Serological investigations, including antinuclear antibodies (ANA) and ANA profile, were negative.

**Figure 7:**
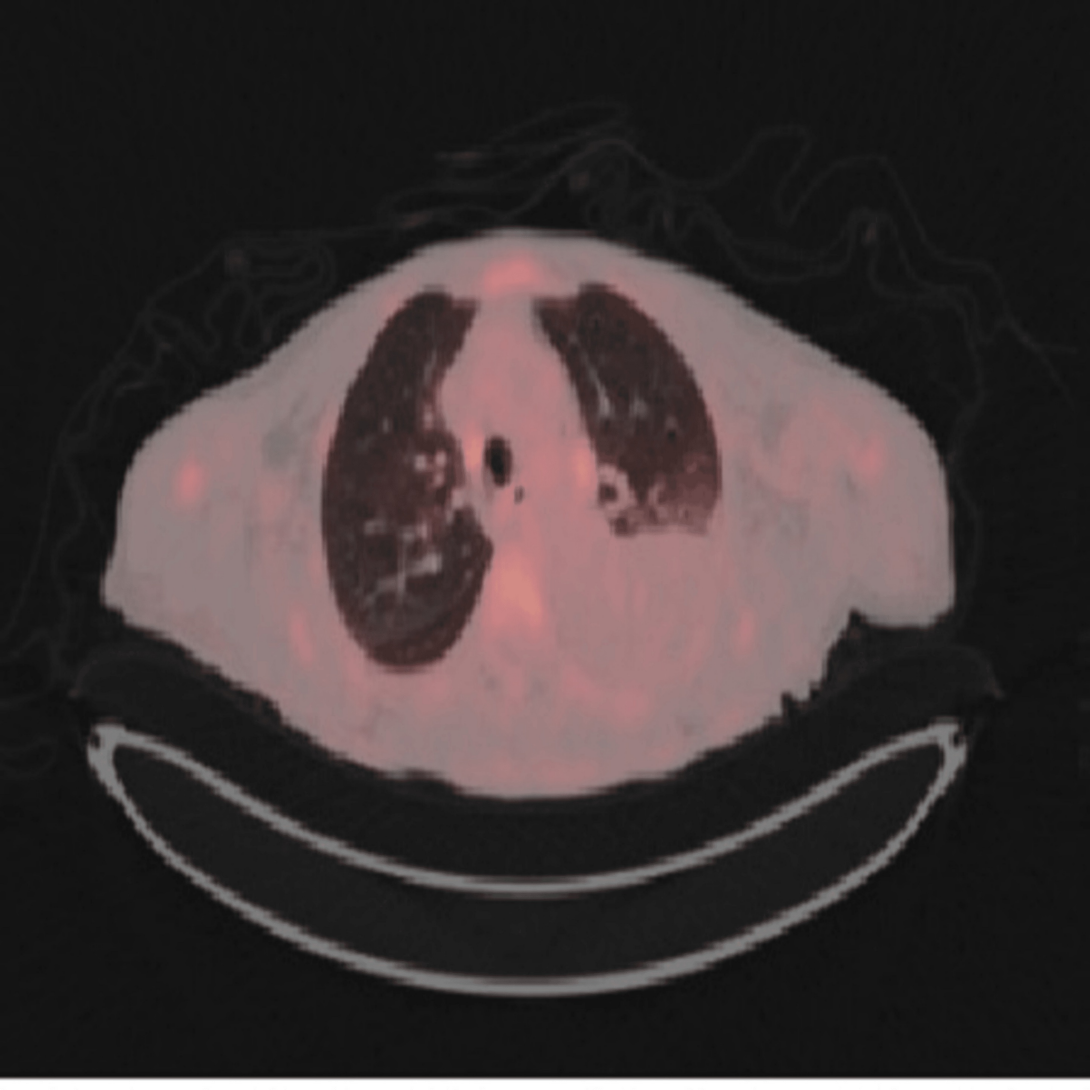
PET scan-thoracic window showing FDG-avid mediastinal lymph nodes. FDG: fluorodeoxyglucose.

A PET-CT was performed, revealing a highly fluorodeoxyglucose (FDG)-avid right vocal cord nodule, FDG-avid mediastinal and porta hepatis lymph nodes (Figure 7). The vocal cord lesion was already known to ENT and under surveillance. Hematology reviewed the case and considered the lymphadenopathy likely reactive; the patient declined lymph node biopsy at that stage.

Nerve conduction studies were carried out, which demonstrated an asymmetric neuropathy with left radial palsy, bilateral ulnar palsy, and possible bilateral peroneal involvement. Electromyography showed severe subacute denervation and a generalized axonal neuropathy, highly suggestive of vasculitic neuropathy. Although interpretation was complicated by edema and some myopathic changes, normal creatine kinase excluded myositis. Clinically, her neuropathy was more pronounced in the lower limbs, producing profound weakness and functional impairment.

**Figure 8:**
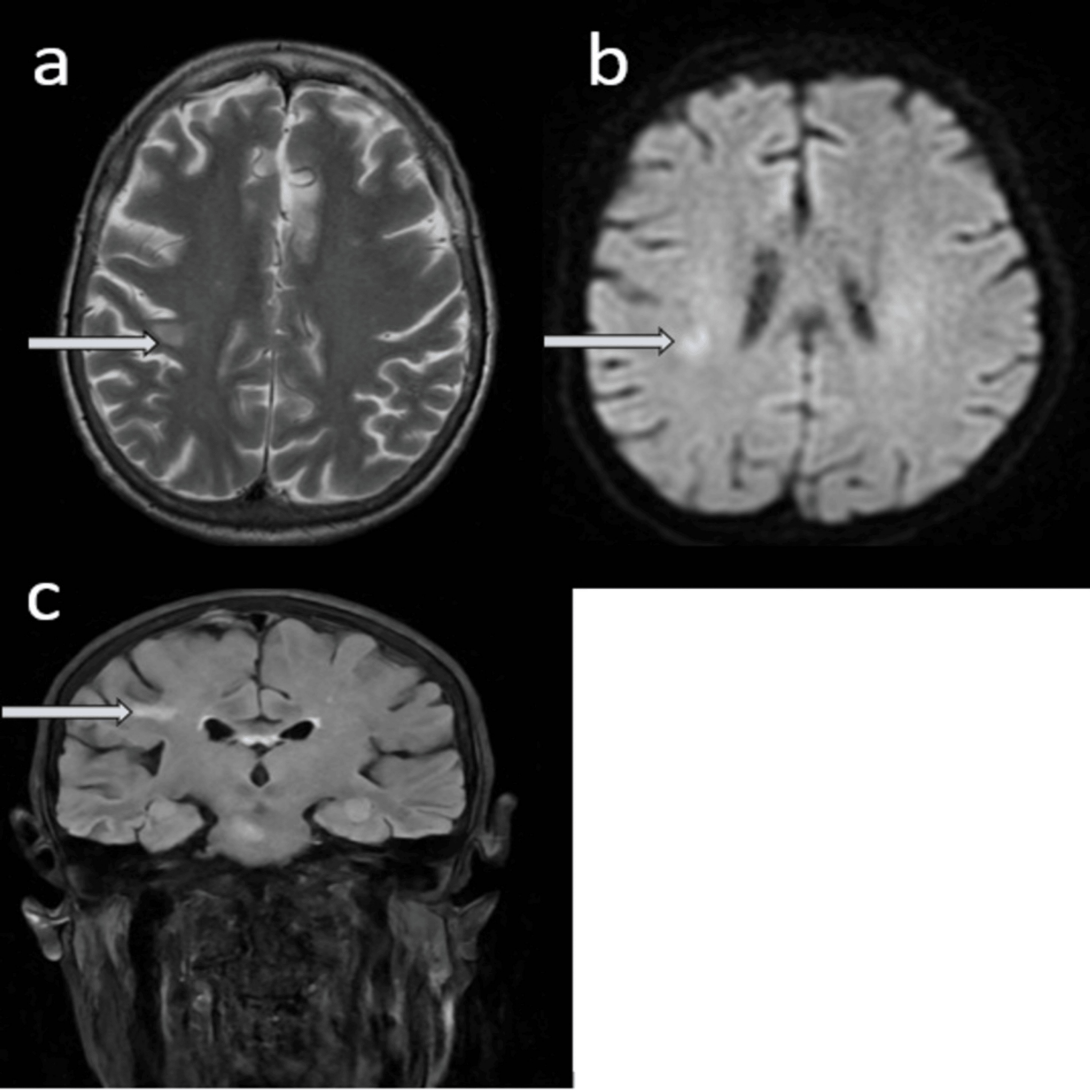
MRI head without contrast showing interval resolution of PRES. (a) T2W image, axial section; (b) DWI image, axial section; (c) FLAIR image, coronal view. DWI: diffusion-weighted imaging; FLAIR: fluid-attenuated inversion recovery.

An MRI of the brain performed later showed interval resolution of the PRES changes (Figure 8), supporting the diagnosis of hypertension-related, reversible encephalopathy. Meanwhile, her systemic features--weight loss, fluctuating confusion, inflammatory arthritis, Raynaud's phenomenon, and pleural and pericardial involvement--all raised suspicion for systemic vasculitis.

**Figure 9:**
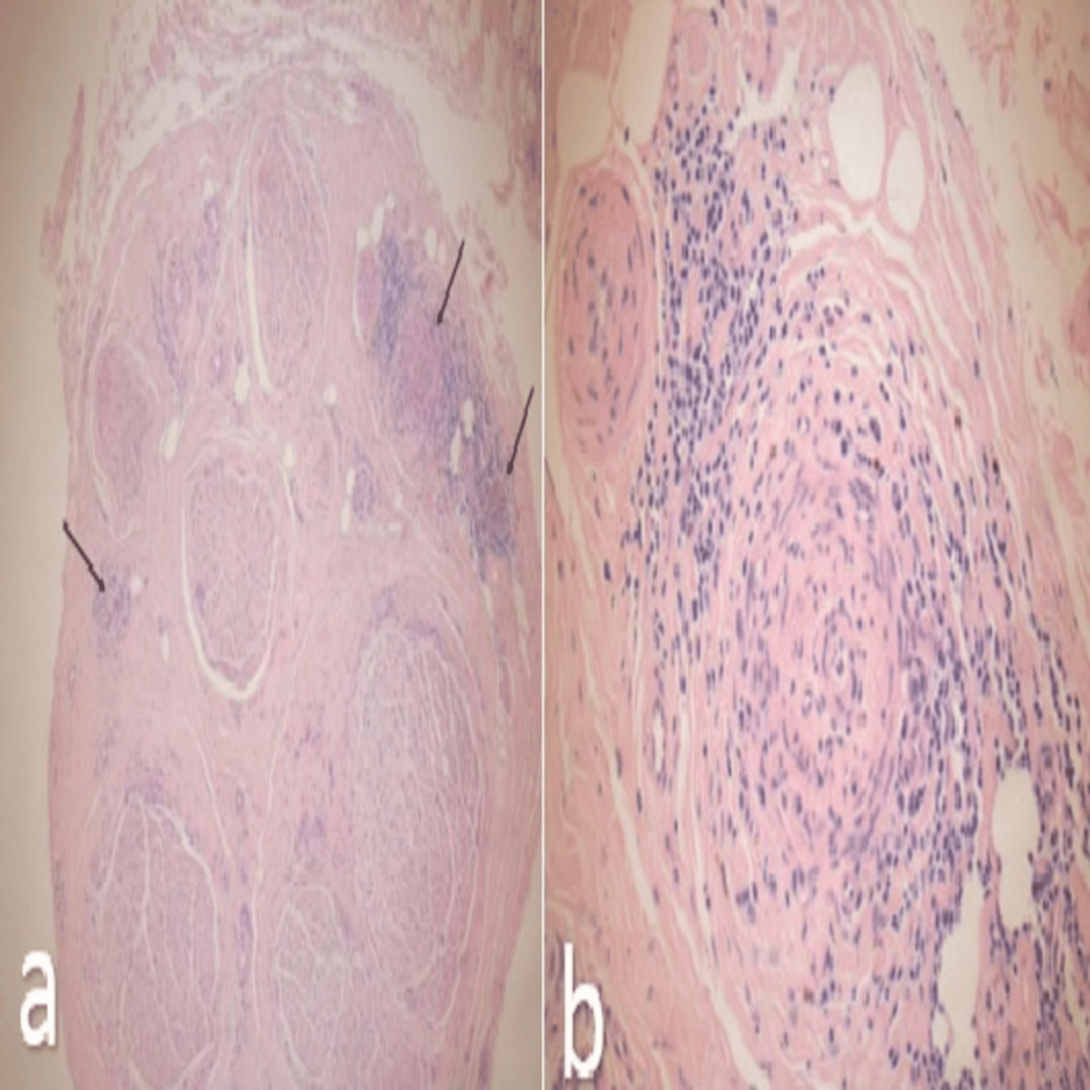
Histopathology of sural nerve biopsy, revealing reduced axonal density and striking perineuritis with evidence of both healed and active vasculitis. (a) Cross-section of nerve showing perineuritis; (b) zoomed in view of the area of active inflammation.

To pursue a definitive diagnosis, a sural nerve biopsy was undertaken. Histopathology revealed reduced axonal density and striking perineuritis with evidence of both healed and active vasculitis (Figure 9). These findings confirmed the diagnosis of vasculitic neuropathy.

Given the biopsy results, the patient was commenced on high-dose prednisolone (60 mg daily). Her seizures did not recur, her blood pressure gradually stabilized, and her neuropathic symptoms stopped progressing. She was subsequently referred to a specialist vasculitis and neurology center for initiation of long-term immunosuppressive therapy and rehabilitation.

## Discussion

This case highlights how systemic vasculitis can first declare itself through simultaneous central and peripheral nervous system involvement, with imaging-confirmed PRES and biopsy-proven vasculitic neuropathy. PRES is thought to arise from failure of cerebrovascular autoregulation under hypertensive stress together with endothelial injury and capillary leak, producing vasogenic edema that is typically reversible when precipitating factors, especially blood pressure elevation and active inflammation, are promptly addressed [7-9,11]. In vasculitis, small case series suggest that PRES may occur at or near disease onset in a minority of patients and can improve with timely hemodynamic control and immunosuppression [10,12]. The peripheral process--vasculitic neuropathy--reflects inflammatory injury of the vasa nervorum with resultant ischemia and axonal degeneration. Clinically, it often presents as a painful, asymmetric sensorimotor neuropathy; electrodiagnostic studies support the diagnosis, but tissue confirmation remains pivotal when non-invasive testing is non-diagnostic [1-4].

The sensitivity of sural nerve biopsy is moderate (≈50-55%), and a combined nerve-muscle biopsy strategy provides a modest incremental yield, a point particularly relevant when multifocal deficits, weight loss, and constitutional features raise the pre-test probability of vasculitis [2,5,6]. However, our patient's diagnosis was established through nerve biopsy alone. Procedural series also emphasize practical aspects, safety, and technique selection for nerve and muscle biopsies in suspected inflammatory neuropathies [13]. The co-occurrence of PRES and vasculitic neuropathy in the same admission underscores a shared pathophysiologic substrate: endothelial injury across vascular beds in both the CNS and peripheral nerves. The parallel radiological resolution of PRES with strict blood-pressure control and early corticosteroid therapy in this patient supports an inflammatory-vascular mechanism consistent with existing literature [7-9,11,12]. From a systems perspective, multidisciplinary coordination among neurology, rheumatology, neuroradiology, and pathology shortens diagnostic latency, increases the likelihood of obtaining diagnostic tissue, and supports timely initiation of immunomodulatory therapy to limit further axonal loss and optimize outcomes [1-4].

## Conclusions

This case underscores several important learning points. Posterior reversible encephalopathy syndrome (PRES) should always be considered in hypertensive patients presenting with seizures, particularly when there are unexplained autoimmune risk factors. In systemic autoimmune diseases, neuropathy frequently warrants further evaluation with biopsy, as electrophysiological studies alone are often insufficient to establish the diagnosis. Nerve biopsy, therefore, remains the diagnostic gold standard for suspected vasculitic neuropathy. In addition, multidisciplinary assessment plays a critical role in the management of complex multisystem inflammatory presentations. Finally, early initiation of immunosuppressive therapy is vital, as it significantly improves long-term neurological outcomes.
